# Climate geroscience: the case for ‘wisdom-inquiry’ science

**DOI:** 10.1098/rsbl.2024.0426

**Published:** 2024-12-11

**Authors:** Colin Farrelly

**Affiliations:** ^1^ Department of Political Studies, Queen’s University Kingston, Ontario K7L 3N6, Canada

**Keywords:** climate, geroscience, science policy, collaboration, ageing, planetary health

## Abstract

Why should, and how can, the fields of climate science and geroscience (which studies the biology of ageing) facilitate the cross-disciplinary collaboration needed to ensure that human and planetary health are both promoted in the future of an older, and warmer, world? Appealing to the ideal of ‘wisdom-oriented’ science (Maxwell 1984 In *From knowledge to wisdom: a revolution in the aims and methods of science*), where scientists consider themselves to be artisans working for the public good, a number of the real-world epistemic constraints on the scientific enterprise are identified. These include communicative frames that stoke intergenerational conflict (rather than solidarity) and treat the ends of planetary and human health as independent ‘sacred values’ (Tetlock 2003 *Trends Cogn. Sci.* **7**, 320–324) rather than as *interdependent* ends. To foster ‘climate geroscience’—the field of knowledge and translational science at the intersection of climate science and geroscience—researchers in both fields are encouraged to think of novel ways they could make researchers from the other field ‘conversationally’ present when framing the aspirations of their respective fields, applying for grant funding and designing their conferences and managing their scientific journals.

## 1. Introduction

The philosopher of science Philip Kitcher argues that ‘responsible biology’ requires much more of scientists than simply engaging in the ethical conduct of research (e.g. not fabricating data) [1]. Scientists should also think of themselves as *artisans* working towards the public good, and as such they ought to reflect upon *the ends*, and not just the means, of their biological research. In *From Knowledge to Wisdom: A Revolution for Science and the Humanities*, Nicholas Maxwell makes a similar point and contends that the scientific enterprise must shift away from the production of ‘knowledge-inquiry’ and move towards ‘wisdom-inquiry’ which takes seriously the question ‘What kind of science, technology, scholarship and education is best designed to help us promote human welfare and realize that which is genuinely of value in life?’ [2].

This opinion aims to help facilitate cross-disciplinary dialogue on responsible biology and ‘wisdom-inquiry’ science by drawing attention to the interdependence of the ends of two areas of biological research that might initially appear, at least at first blush, to be pursuing potentially conflicting ends—climate science and geroscience (which studies the biology of ageing). Climate science studies the Earth’s climate in the hope that doing so will lead to translational science that helps with the mitigation of, as well as adaptation to, climate change to improve planetary (including human) health. Geroscience is a multidisciplinary approach to the study of the biology of ageing, examining the genetic, environmental, dietary and pharmacological factors which influence the onset of disease, frailty and disability in late life. The aspirational goal of geroscience, analogous in ambition to climate science’s carbon ‘net zero’, is known as the *Geroscience Hypothesis* [3,4]—the conjecture that translational gerontology can target the mechanisms of biological ageing to extend a *healthy* lifespan.

Geroscience has recently begun to explore the feasibility of ‘gerotherapeutics’, drugs that target pathways involved in ageing with the aim of reducing the burden of ageing-related diseases and increasing healthspan and lifespan [5,6]. Unlike exercise, improved nutrition or connecting with nature, which can improve mental and physical health but are not typically considered to conflict with the aims of climate science, this Opinion focuses on the still experimental aspects of translational gerontology that aspires to develop drugs to intentionally target the mechanisms of ageing to increase healthspan and lifespan. That is the element of geroscience research most likely to be perceived as conflicting with climate science, but may in fact yield important interventions for both the mitigation of, and adaptation to, climate change.

The *UN Decade of Healthy Aging: Plan of Action* (2021–2030) urges change in the way we think, feel and act towards age and ageing, as well as greater collaboration to address the harmful effects of climate change on older persons [7]. Haq & Gutman [8] make a compelling case for what they call ‘climate gerontology’, research at the intersection of climate change and population ageing. This opinion hopes to add to their call for greater cross-disciplinary collaboration, with a specific focus on the overlap between climate science and geroscience that focuses on developing pharmacological innovations to target the mechanisms of ageing. I call this more specialized focus on the intersection of climate science and geroscience ‘climate geroscience’. I contend that when climate science embraces narratives of *intergenerational solidarity*, and geroscience an *ethos of planetary health stewardship*, fruitful avenues of overlap between the two fields, in terms of translational science, are revealed. This domain of cross-disciplinary knowledge is the focus of climate geroscience.

## 2. Why think there is tension between these two fields of science?

NASA models estimate that, depending on the action taken to reduce greenhouse gases, the global temperature can be expected to rise between 2.5**°**C and 4.5**°**C by the year 2100 [9]. And the United Nations estimates that global life expectancy at birth in the year 2100 will rise (from its current 73 years of life) to 82.6 years [10]. Climate change, and the collective efforts to mitigate the health, ecological and economic harms of a warming planet, represents a major challenge for climatology, communication science and global policymaking. What makes these challenges even more Herculean is the empirical reality that the predicament of climate change is not occurring in isolation from other important global developments. Global warming is occurring *simultaneously* with novel and significant societal and technological transformations, including population ageing and advances in translational gerontology. The latter refers to the application of discoveries from the basic biology of ageing to novel public health interventions that could increase both life expectancy and healthspan at the individual and population levels [11]. And this is where the commonly perceived tension between these two areas of science typically arises.

**Figure 1.**
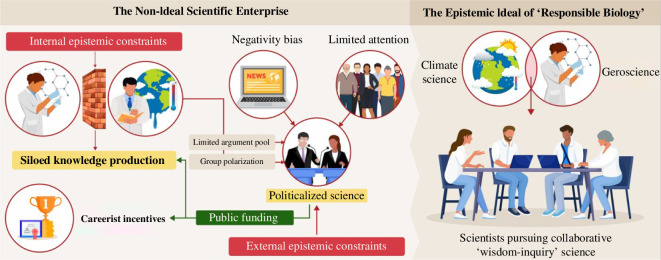
Visual representation of the gap between the ‘non-ideal’ scientific enterprise and the ideal of ‘wisdom-inquiry’ science.

Population growth and human activities, such as agriculture and the use of fossil fuels, have been the main drivers of climate change. The suggestion that science should also harness knowledge about the biology of ageing to potentially *increase* the amount of time the global population survives might initially sound contradictory to the ideal of responsible biology. However, the common (mis)perception that the ends of climate science and geroscience are in tension or conflict arises because of some of the communicative frames utilized by researchers functioning within the ‘siloed’ knowledge production of the scientific enterprise, an enterprise where science is often politicalized [12] and the audiences scientists are trying to reach (i.e. the media, general public and policy makers) have a limited attention span [13]. Furthermore, most people learn about science through the ‘mediated realities’ of offline and online news [14], and negativity drives online news consumption [15] (figure 1). Some of these communicative frames and epistemic distortions could be remedied by greater mutual understanding and collaboration between climate science and geroscience.

The following are three common (but mistaken) presumptions of the ‘conflictual’ interpretation of climate science and geroscience:

1. Framing climate change as a problem of intergenerational *conflict*.
2. Framing the goal of carbon ‘net zero’ as a ‘sacred value’.
3. Framing the ends of geroscience in unrealistic terms—such as ‘conquering aging’ or ‘radical life extension’—thus giving the impression the field lacks an ethos of planetary health stewardship.

In communication science, a ‘frame’ refers to a communication that ‘organizes everyday reality’ [16]. Frames are cognitive constructs that can be ‘agenda setting’ for current affairs by identifying or amplifying certain information, problems or potential remedies and bracketing or ignoring others. By encouraging more researchers to work at the intersection of ‘climate geroscience’, climate science and geroscience can refine the framing of the public communication of their respective fields of science to ensure they do not marginalize or bracket the significance of findings in the other field, which could jeopardize ‘wisdom-inquiry’ science.

Climate change is commonly framed as a problem of intergenerational wellbeing [17], with older persons (especially those in the developed world) characterized as ‘climate change culprits’—those most responsible for the climate crisis because of their past energy consumption and their failure to act sooner to reduce greenhouse gas emissions. By contrast, younger persons (as well as future generations) are portrayed as the ‘victims’ of climate change, those who must bear the brunt of the ecological, economical and health burdens of climate change [18]. A focus on guilt and moral responsibility for climate change has been emphasized by some scientists [19] and climate activists as a way to increase support for climate change policies.

Adopting the frame of intergenerational conflict for climate change may increase the guilt that garners support for action on climate change, but it also has deleterious effects on intergenerational solidarity, as well as the factual accuracy of the harms of climate change. The political influence environmental science and climate activists enjoy today was forged through decades of political action and efforts from (now older) scientists and activists, like those who first observed Earth Day in 1970, or founded Greenpeace in 1971. Furthermore, older persons are among those most vulnerable to the harms of climate change [20–26]. Today’s youth will age on a warmer planet, and thus they too will face the health vulnerabilities of ageing. However, these vulnerabilities will be amplified on a warmer planet, which is why the science of healthy ageing is critical to their future health prospects, as well as to the health of future generations.

A second common misconception about the alleged conflict between the ends of climate science and geroscience is sometimes stoked by advocates of both fields of science when, in an effort to boost support for their respective causes, they characterize the values of the field’s ends as ‘sacred values’. The term ‘sacred values’ was coined by the psychologist Phil Tetlock [27], who defines a value as ‘sacred’ when it is considered absolute and inviolable, something that cannot be reasonably traded off against other values (like monetary gain). When climate scientists and geroscientists address their respective fields in the ‘siloed’ approach of ‘knowledge-inquiry’, they risk engaging in the ‘limited argument pool’ that leads to group polarization (i.e. becoming more extreme in their position) [28]. In other words, the complementary values of planetary health and human health might be construed as independent ‘sacred values’ by some in a siloed ‘knowledge-inquiry’ approach to translational science.

For example, climate science is committed to pursuing the mitigation strategy of carbon ‘net-zero’. Population growth and human activities have been the main drivers of climate change. With the United Nations estimating the global population to rise to over 11 billion people by the end of this century, one might form the opinion that the development of gerotherapeutics which increase (healthy) lifespan would pose an unacceptable risk to planetary health because it may expand, rather than reduce, overall greenhouse gas emissions.

However, while the goal of ‘net-zero’ is a morally laudable scientific aspiration for climate science, it is not meant to be treated as a ‘sacred value’ that trumps human health. Instead, it is a prime example of the importance of ‘wisdom-inquiry’ science, as it is a goal that requires numerous ethical judgements, and raises diverse social concerns and economic considerations [29]. But if the only value that mattered was planetary health, then *all* of medical science, from sanitation science to immunizations, should be forfeited to reduce the ecological impact of human populations by trying to reduce the size of the latter to as small a number as possible. Such a suggestion would of course be morally abhorrent. Population growth and population ageing are not demographic trends to catastrophize, they are a reflection of the reality that the world’s historically high extrinsic mortality risks have been abated. Forfeiting public health measures to reduce greenhouse gas emissions is not a justifiable *means* of achieving ‘net-zero’. ‘Net-zero’ is a laudable scientific aspiration when it promotes both human and planetary health.

What those who object to, or raise concerns about, increasing lifespan (rather than healthy lifespan) typically have in mind is the concern that an extension of the period of the lifespan when adults in advanced ages are in poor health—managing multi-morbidity/frailty/disability—may not be morally desirable or a reasonable trade-off with planetary health. This is a more nuanced and complex challenge to address [30], which I do not have space to explore here. But that specific worry is not one that should be expressed to oppose the development of gerotherapeutics. The latter are intended to ‘add life to years, not (unhealthy) years to life’ (though additional years of life is likely to be a consequence of extended healthspan). Improving the biological resilience of human populations, whether it be through drug development to alter the rate of ageing or traditional preventative health measures like nutrition, exercise, or equitable access to the social determinates of health (e.g. economic security, education, etc.), are all part of the adaptation strategy to climate change because a warmer planet will exacerbate the health risks of vulnerable populations.

Geroscience advocacy is also susceptible to framing distortions when advocates invoke aspirations like ‘defeating aging’ [31,32] with little to no regard for planetary health. Such frames can distract ‘wisdom-inquiry’ science from the real important questions to address, such as the *interdependence* of human and planetary health when they presume that geroscience represents a ‘magic bullet’ to healthy longevity (regardless of the state of planetary health). Frames that invoke talk of ‘radical life extension’ or ‘biological immortality’ not only contravene the scientific credibility of geroscience (the rate of ageing is malleable, but that does not mean that ageing can be eliminated), they can create the impression that human health is independent of, rather than interdependent with, planetary health. Climate geroscience encourages geroscientists to conceptualize translational gerontology as a tool of planetary health stewardship, something that could help advance both healthier environments and healthier (older) populations.

## 3. The environment and ageing

An extensive amount of research has documented how the environments human populations live in impact health, over the lifespan, by influencing the rate of biological ageing. Air pollution is associated with higher epigenetic ageing [33], groups exposed to racialized, economic and environmental injustice may face elevated morbidity and mortality risks from accelerated ageing [34], and lifestyle variables (e.g. alcohol consumption, smoking, physical activity and social integration) can increase the risks of suffering from structural brain atrophy [35]. Healthy ageing, whether through improved nutrition, exercise, access to the social determinates of health or novel gerotherapeutics, should be conceptualized as an integral part of the mitigation and adaptation strategies of climate science.

With respect to reducing greenhouse gas emissions, shrinking the gap between ‘life expectancy’ and ‘healthy life expectancy’—by increasing the latter—would bring significant economic benefits [36], and a reduction in both the healthcare expenditures for ageing populations as well as the greenhouse gas emissions from healthcare systems. Some estimates suggest that the healthcare sector is responsible for approx. 4.4% of the world’s total carbon emissions [37,38]. Improving the health of older populations, by preventing and delaying many of the chronic diseases of late life, would reduce these emissions. It is also worth noting that if biological ageing is *not* slowed, and medical science continues down the path of trying to prevent death from each chronic disease in late life, older populations will survive longer managing chronic disease and frailty, including more debilitating conditions. As Olshansky has noted, finding a cure for cancer may cause an unintended increase in the prevalence of Alzheimer’s disease [39]. This would not be a desirable outcome from the perspective of either human or planetary health.

Translational gerontology may also help reduce the carbon emissions from non-human animals, such as agricultural and companion animals. NASA estimates that the concentration of methane in the atmosphere has more than doubled over the past 200 years and is responsible for 20–30% of the global warming that has occurred since the Industrial Revolution [40]. Agriculture is one of the major sources of methane. The ageing and longevity of agricultural mammals like cows, pigs, goats and sheep directly impact the amount of greenhouse gases emitted from agriculture. Translational gerontology could potentially impact the productivity and lifespan of some agricultural mammals in ways that help reduce the emission of carbon that contributes to climate change. Dairy cattle are typically slaughtered for meat once their milk production diminishes. The estimated maximum lifespan of a dairy cow is about 20 years [41]. However, the average productive lifespan is approx. 3–4 years in countries with high-producing dairy cows [42]. Once their ability to produce milk diminishes, dairy cattle are slaughtered for meat. This disparity between their lifespan and ‘productive lifespan’ means that many more dairy cattle are bred and raised to produce the same amount of dairy products that could be yielded by a smaller number of dairy cows if their productive lifespan approximated the maximal lifespan. Insights from translational gerontology could lead to interventions that help reduce the amount of greenhouse emissions from agricultural mammals by extending the period of health and productivity.

The ‘Dog Aging Project’ [43] is an example of how translational gerontology might help reduce the gap between lifespan and healthspan for companion animals. It is a long-term longitudinal study, involving tens of thousands of companion dogs, to identify the genetic, environmental and lifestyle factors associated with a healthy lifespan [44]. One study the Dog Aging Project is currently conducting is ‘Test of Rapamycin In Aging Dogs’ (TRAID), which involves assessing whether the consumption of the molecule rapamycin, which increases lifespan and healthspan of mice [45,46], has a similar effect in dogs. Such a study provides not only useful information for developing drug interventions in humans, but it may also help promote planetary health by reducing some of the carbon emissions from pet ownership.

The environmental impact of the life cycle of pet dogs in the European Union is estimated to be around 7% of the annual climate change impact of an average EU citizen, caused primarily through pet food. US dogs and cats consume as much dietary energy as ~62 million Americans, approximately one-fifth of the US population [47]. If ‘net zero’ is conceptualized as a ‘sacred value’ then one might conclude that pet ownership is morally impermissible, because of these environmental impacts. But pet ownership can serve important, morally laudable, aims, such as improving the physical and psychology wellbeing of owners [48]. As such it is an interest that must be balanced against the goal of ‘net zero’.

Larger breeds of dogs have a shorter lifespan—by about 5 years on average and up to 8 years—[49] compared to smaller dogs, and larger breeds consume more food which contributes to climate change. If, as may be the case for at least many pet owners, smaller breeds of dogs can confer comparable levels of physical and psychological benefits to their owners—such as exercise and the provision of social support— there are ecological reasons for prioritizing the ownership of smaller breeds of dogs. Furthermore, because of their faster metabolism and rapid growth and development, puppies require more food than adult dogs. And feeding quantities *reduce* as dogs get older. The feeding regime for senior dogs is estimated to be 20% lower than that of younger adult dogs [50]. Extending the healthy lifespan of dogs could conceivably reduce some portion of the carbon emissions from pet food if increasing the healthy lifespan of dogs would provide more years of pet companionship with less food consumption (and thus environmental impact) compared to the same quantity of pet companionship achieved via increased numbers of puppies reared for pet companionship.

The costs for, and greenhouse gas emissions from, healthcare and agricultural and companion animals could potentially be reduced by interventions from translational gerontology. When extended life is *healthy life*, it is good for both human and planetary health. Financial savings from a reduction in frailty, disability and disease in late life could be utilized to invest more heavily in the mitigation of, and adaptation to, climate change. This will be particularly important for lower- and middle-income countries that have larger ageing populations (especially China and India). Climate geroscience should frame the economic benefits accrued through healthy ageing as something that ought to be re-invested, for both intergenerational solidarity and planetary health, in mitigation and adaptation measures to benefit the future of humanity and the planet.

## 4. Conclusion

The climate scientists of the future should theorize about solutions for mitigation and adaptation knowing that the future will be one with older populations. And the geroscientists of the future should theorize about solutions to age-related health challenges knowing that the future will be warmer than it is today and that planetary health is interdependent with human health. But the ‘siloed’ pursuit of scientific knowledge can impede the pursuit of the ‘wisdom-inquiry’ science required to meet the challenges of an ageing and warming world. Climate geroscience encourages greater understanding of, and collaboration between, climate science and geroscience. Some feasible, concrete measures that could be taken to help gain momentum in this direction would be to increase the opportunity for scientists in both fields to be ‘conversationally present’ with each other. Scientific conferences in climate science and geroscience might allocate specific panels to deal with issues that arise at the intersection of the two fields, and/or include discussants from the other field on some panels to help redress the impact of limited argument pools and group polarization.

**Figure 2.**
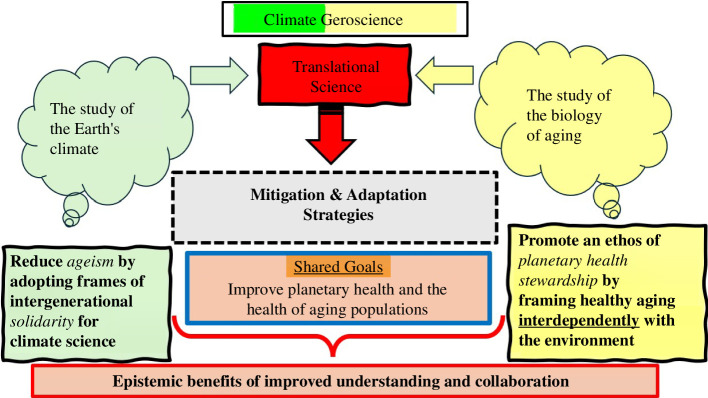
The epistemic benefits of cross-disciplinary collaboration.

Funding agencies could require grant applicants to include some details of how translational science in either field could foster both intergenerational solidarity and planetary health. Journals can invite editorials and opinion submissions from leading scientists in the other fields to offer insights on what they see as the challenges and opportunities for translational science, from an ‘outsider’s perspective’. And scientific associations in both fields could play a formative role in encouraging greater collaboration by surveying their members to ask for feedback on their member’s perceptions of the aspirations of the other field of science. For example, do most climate scientists think slowing human ageing would be desirable, if it extends healthy life expectancy? Why/why not? And what do geroscientists think the ecological impact of slowing human ageing will be? This could help abate the politicalization of science, which often leads advocates of both areas of science to adopt frames of tension and conflict which marginalize climate geroscience (figure 2).

## Data Availability

This article has no additional data.
